# Respiration and Heart Rate Monitoring in Smart Homes: An Angular-Free Approach with an FMCW Radar

**DOI:** 10.3390/s24082448

**Published:** 2024-04-11

**Authors:** Pouya Mehrjouseresht, Reda El Hail, Peter Karsmakers, Dominique M. M.-P. Schreurs

**Affiliations:** 1 Waves: Core Research and Engineering (WaveCoRE), Department of Electrical Engineering (ESAT), KU Leuven, B-3001 Leuven, Belgium; dominique.schreurs@kuleuven.be; 2 Division of Declarative Languages and Artificial Intelligence (DTAI), Leuven.AI, Department of Computer Science, KU Leuven, B-2440 Geel, Belgium; reda.elhail@kuleuven.be (R.E.H.); peter.karsmakers@kuleuven.be (P.K.); 3 Flanders Make, MPRO, B-3000 Leuven, Belgium

**Keywords:** beamforming, FMCW radar, smart homes, two-dimensional localization, vital signs, wide angular monitoring

## Abstract

This paper proposes a new approach for wide angle monitoring of vital signs in smart home applications. The person is tracked using an indoor radar. Upon detecting the person to be static, the radar automatically focuses its beam on that location, and subsequently breathing and heart rates are extracted from the reflected signals using continuous wavelet transform (CWT) analysis. In this way, leveraging the radar’s on-chip processor enables real-time monitoring of vital signs across varying angles. In our experiment, we employ a commercial multi-input multi-output (MIMO) millimeter-wave FMCW radar to monitor vital signs within a range of 1.15 to 2.3 m and an angular span of −44.8 to +44.8 deg. In the Bland–Altman plot, the measured results indicate the average difference of −1.5 and 0.06 beats per minute (BPM) relative to the reference for heart rate and breathing rate, respectively.

## 1. Introduction

Smart home technologies have revolutionized the way we interact with our living spaces, offering convenience, automation, and an enhanced quality of life. Beyond traditional applications like lighting control and security systems, smart homes are increasingly being explored for healthcare and wellness monitoring using radars [1,2,3,4,5,6,7,8]. This technology can monitor vital signs such as heart rate and respiratory rate remotely and in a non-invasive way, providing valuable data for healthcare individuals. Among different types of radars, the Frequency Modulated Continuous Wave (FMCW) technique has been widely used to monitor vital signs because of good range and velocity resolution, high SNR, and simple and low-cost structure [1].

Detecting vital signs using radars is based on analyzing reflected signals from the target, a person. The oscillation of the thoracic region and the rhythmic contraction of the heart alters the signal incoming to the organism, and applying signal processing algorithms to the reflected signal can reveal information about physiological parameters. Therefore, the person should expose their chest to the transmitted signal for the radar to accurately detect vital signs. This can be achieved by standing in front of the radar sensor or lying down on a bed equipped with radar technology on the ceiling. In the majority of works, the individual in question assumes an anterior position to the radar without any angular orientation [9,10,11,12,13,14,15]. The ability to monitor vital signs from various angular positions presents a significant advantage in the context of smart home applications. This advantage eliminates the necessity for individuals to be directly positioned in front of the radar system [16,17,18,19,20,21,22], which allows greater flexibility and convenience as individuals can perform their daily activities while still having their vital signs monitored. Additionally, this advantage enhances the usability and effectiveness of smart home applications in monitoring and maintaining individuals’ health and well-being. However, some other practical aspects should be considered to pave the way for realizing a healthcare radar-based system [1]. One aspect is that monitoring vital signs should be applicable in a real-time manner. In other words, it is necessary for the computational effort to be minimized to facilitate implementation on hardware with low complexity. The second is cost, which can play an important role in smart homes. It is clear that using commercial radars which are available in the market at a reasonable price is more advantageous than becoming involved in designing special radars for this purpose, which may result in a bulky and complex structure. Additionally, these products undergo testing in accordance with safety regulations, ensuring their suitability and safety for indoor use [23]. The third factor is power consumption, contributing to a more sustainable and cost-effective smart home. Finally, it is worth mentioning that a radar can detect not only physiological parameters but also people’s location [20]. The detected location helps ease automated controls in smart homes. For example, temperature and lights can be controlled according to the person’s presence in different locations in the smart home. Therefore, an efficient approach is to use the full potential of the radar and integrate the various functions to realize a multi-functional system.

As mentioned, in most papers, it is assumed that the living subject is in front of the radar (without any angular position). In the work conducted by authors in [10,11,12], commercial FMCW radars were employed to extract physiological indicators from varying distances of up to 2.5 m, without angular orientation and with offline processing. In the study outlined in [14,15], measurements were conducted to monitor vital signs of an individual who lay on a bed in front of the radar. However, in [16], a tailored FMCW radar system was utilized to realize breathing and heart rate monitoring in an online manner and using 1D localization. The radar system was capable of detecting these physiological parameters at a distance of 1 m, even when the subject’s angular orientation was varied by up to 30 degrees. Likewise, an FMCW radar with an 8 W power consumption was designed in [18] to detect heart and breathing rates from a distance of up to 5 m and different angles (up to 37 degrees at 1 m distance) by offline processing. In [20], a specially designed FMCW radar that incorporated two frequency scanning antennas was proposed for 2D localization and detecting the physiological parameters in a real-time process. The monitoring was carried out at different distances, specifically within the range of 2 to 4 m, and at varying angles spanning from 0 to 45 degrees. In [22], a specially designed Doppler radar was utilized to identify the presence of an individual within a room and subsequently track their vital signs at periodic snapshots when the person was in different locations.

Although valuable, the methods in the aforementioned works are either not able to perform angular monitoring or disregard or partially address the practical considerations. These shortcomings are important limitations in smart home applications. Recognizing these limitations, our conference paper [21] laid the groundwork by exploring the feasibility of respiration and heart rate monitoring across various angular orientations, showcasing preliminary findings that highlighted the potential of this approach. In this paper, we delve into refining our methodologies to enhance the accuracy of vital sign detection and substantially broaden the effective detection range. To achieve real-time processing, some parts of the proposed vital sign algorithm are processed by the on-chip processor embedded in the radar to decrease computational complexity. Also, a time-frequency analysis technique, specifically continuous wavelet transform (CWT), is suggested to extract vital signs frequencies. In comparison to other techniques such as adaptive decomposition algorithms [10,12], Fast Fourier transform (FFT) [9,14], and independent component analysis (ICA) [15,24], this technique allows for capturing events of different durations and frequencies in the radar data. Unlike FFT, which analyzes frequency under the assumption that the signal is stationary, CWT can identify and localize transient features and temporal variations in non-stationary signals where frequency components fluctuate over time.

The structure of this paper is organized as follows: Section 2 introduces our proposed concept along with the algorithm for vital sign detection. Section 3 details the simulation process of the algorithm. Section 4 outlines the initial steps involved in our proposed methodology. Section 5 discusses the obtained measurement results. Finally, Section 6 concludes the paper.

## 2. Proposed Approach: Capturing Vital Signs across Wide Angles

**Figure 1:**
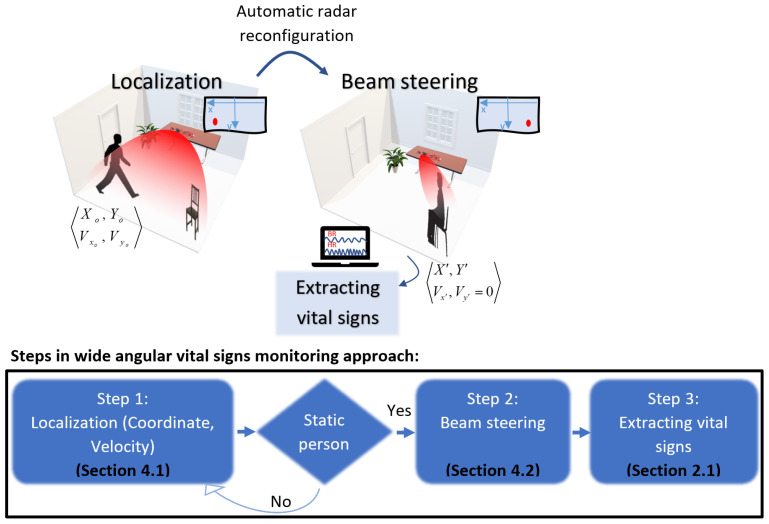
The proposed approach for wide angular vital signs monitoring: Tracking the person’s location ($X_{o}$, $Y_{o}$) and velocity ($V_{x_{o}}$, $V_{y_{o}}$), and next focusing the radar beam to the static location (*X*′, *Y*′) for extracting breathing (BR) and heart rate (HR).

Figure 1 illustrates the suggested approach for monitoring vital signs over a wide angular range using a three-step process. Step 1 involves detecting the presence and movement of a person within the environment. The radar captures both the spatial coordinates ($X_{o}$, $Y_{o}$) and the velocity ($V_{x_{o}}$, $V_{y_{o}}$) of the individual. This is important for determining the direction in which the radar’s beam should be focused for accurate vital sign monitoring. We use the suggested algorithm in [25]. The technical details of this process are elaborated in Section 4.1 for the process completeness.

In Step 2, upon confirming the presence of a static person, the radar is automatically reconfigured to direct the beam towards the detected individual. This is critical for isolating the person’s signal from any background noise or interference and ensuring that the vital signs are accurately captured. This dynamic adjustment capability of the radar is detailed in Section 4.2.

With the beam correctly oriented, heart rate (HR) and breathing rate (BR) are extracted using a suggested processing pipeline. This final step is thoroughly discussed in Section 2.1. Some of the signal processing in this step is performed utilizing the radar’s internal processor.

### 2.1. The Processing Pipeline for Extracting Vital Signs

**Figure 2:**
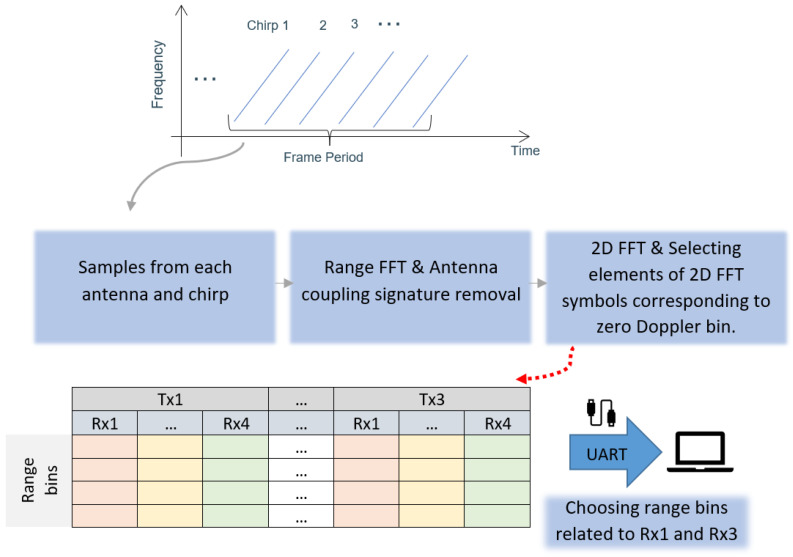
Radar processing pipeline for monitoring vital signs, processed by the radar’s internal processor. The range bins are sent via Universal Asynchronous Receiver–Transmitter (UART).

**Figure 3:**
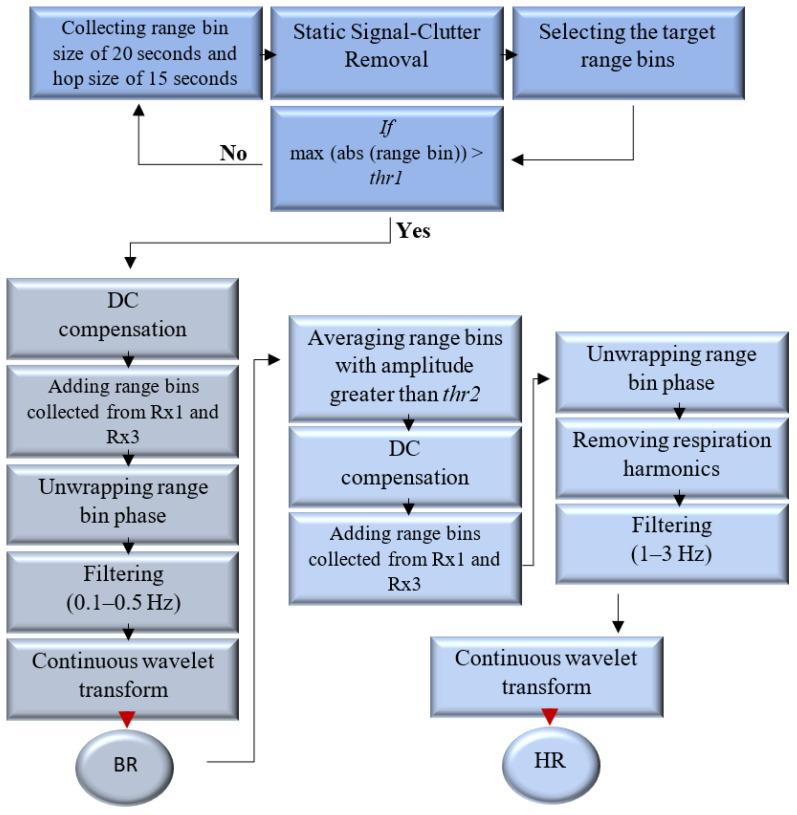
Vital sign detection algorithm.

The FMCW radar transmits a continuous wave signal. The frequency of this transmitted signal is continuously modulated over time in a linear manner, which is often referred to as a “chirp”. The radar employs multiple chirps transmitted over a frame period and collects samples from each receiver antenna. On the received samples, the data processing steps include range FFT and antenna coupling signature removal. Consequently, a matrix representing range bins for each chirp and each receiver antenna and transmitter antenna combination is created. This matrix is then subjected to a 2D FFT, which highlights the elements corresponding to the zero Doppler bin. These selected elements are then organized into separate tables for each receiver–transmitter antenna pair, determining the relevant data for further evaluation. All these processes are carried out by the built-in processor in the radar. Figure 2 illustrates the described pipeline. As seen, the processed results are then transmitted to a laptop for further data processing and visualization scheme for vital sign monitoring. The selected FMCW radar system, specifically IWR6843AOPEVM, Texas Instrument, Dallas, TX, USA, comprises three transmitter antennas and four receiver channels. After the radar’s internal processor processes the reflected signals, the algorithm, depicted in Figure 3, is utilized to detect vital signs. The range bins are collected using a sliding window with size of 20 s and hop size of 15 s to generate a matrix named slow-time matrix. In the subsections that follow, the steps are explained in more detail. This algorithm distinguishes itself from the one proposed in [21] by implementing a static signal clutter removal technique. It incorporates a threshold, denoted as thr1 in Figure 3, to eliminate undesired signals. Additionally, it utilizes two distinct processing pipelines and frequency filtering techniques to detect breathing and heart rate, respectively. In the heart rate detection pipeline, another threshold, thr2 (as depicted in Figure 3), is applied to process the range bins adjacent to the target bin. This involves averaging the data from 10 consecutive indexes surrounding the target bin, thereby enhancing the signal strength. The determination of thr1 and thr2 is empirical.

#### 2.1.1. Static Signal Clutter Elimination and Target Range Bin Allocation

**Figure 4:**
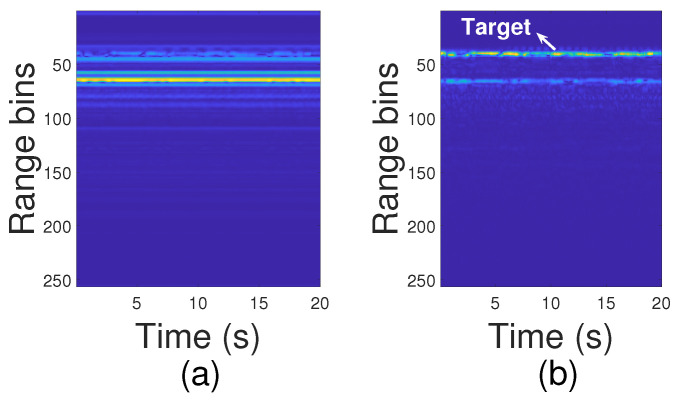
Slow-time matrix: (**a**) before removing static clutters; (**b**) after removing static clutters.

Although the target distance and consequently the corresponding range bin are known thanks to the localization algorithm, selecting the target range bin is ambiguous because of reflected static clutter signals. The interference of these signals is caused by the presence of a complicated background. Not only does it affect the target range bin selection, but it also generates unwanted phase noise, which makes extracting vital information more difficult. Therefore, it becomes essential to reduce the impact of static clutter. The clutter signals coming from other stationary items in a given test situation typically do not change much over slow time. Hence, the background noise can be eliminated by subtracting the mean value of each range bin in the slow-time window from the slow-time matrix. We let *M* be a matrix wherein the rows signify the range bins and the columns denote all the captured range bins per frame. The matrix obtained after the elimination of static clutters can be expressed as follows:

(1) $$M^{\prime }\left(m,l\right)=M\left(m,l\right)-\frac{1}{L}\sum_{l^{\prime }=1}^{L}M(m,l^{\prime })$$

where $m=1,2,\ldots ,m_{rangebins}$, l=1,2,…,L are the indexes of range bins and frames, respectively. Figure 4 shows the matrix before and after applying the static clutter elimination. As seen, the target range bins can be recognized clearly after removing static clutters.

#### 2.1.2. DC Compensation and Unwrapping Phase

The presence of DC interference can have an important effect on the precision of extracting vital signs. Environmental factors and sensor-related issues can all generate DC interference, which can appear as a constant voltage offset in the acquired signals, obscuring the vital sign data that is desired. Hence, DC compensation techniques are utilized to mitigate the impacts of the DC interference. The primary objective of the DC compensation is to eliminate or reduce the DC component of acquired signals while preserving the physiologic variations of interest. According to [14], the problem is a linear least square estimator (LLSE), as follows:

(2) $$\begin{array}{c} \underset{\;\;\;\;Y}{\;\;\min}\;{∥\Lambda Y-B∥}^{2}\\ subject\;to:\;\;{∥\left[\begin{array}{c} x_{O}\\ y_{O} \end{array}\right]∥}^{2}-\Gamma \;>\;0\; \end{array}$$

where

(3) $$\begin{array}{c} \Lambda =\left[\begin{array}{cc} \begin{array}{c} 1\\ \vdots \\ 1 \end{array} & \begin{array}{c} -2{\left[R_{1}\;Q_{1}\right]}^{T}\\ \vdots \\ -2{\left[R_{N}\;Q_{N}\right]}^{T} \end{array} \end{array}\right]\\ B=\left[\begin{array}{c} -{∥\left[R_{1}\;Q_{1}\right]∥}^{2}\\ \vdots \\ -{∥\left[R_{N}\;Q_{N}\right]∥}^{2} \end{array}\right]\\ Y={\left[\begin{array}{cc} \Gamma & {\left[\begin{array}{c} x_{O}\\ y_{O} \end{array}\right]}^{T} \end{array}\right]}^{T}\\ \Gamma ={∥\left[\begin{array}{c} x_{O}\\ y_{O} \end{array}\right]∥}^{2}-r^{2} \end{array}$$

In (3), $R_{N}$ and $Q_{N}$ are the real and imaginary parts of range bins, *N* is the number of range bins, $x_{O}$, $y_{O}$ and *r* are the hypothetical center and radius of the constellation. ${\left[.\right]}^{T}$ is the transpose operator.

After compensation, to increase the signal-to-noise ratio (SNR), the summation of the target range bins from different receiver channels is used to extract the signal phase by the four-quadrant inverse tangent. Since the phase varies between −π and π, there are discontinuities in the extracted phase. This can be tackled by using an unwrapping algorithm. In this algorithm, whenever the transition between successive angles exceeds or equals π, the angles are modified by incorporating multiples of ±2π until the transition is less than π.

#### 2.1.3. Time-Frequency Analysis: Respiration and Heart Rate Estimation

Before applying time-frequency analysis, specifically CWT, a bandpass filter is used with cut-off frequencies of 0.1 Hz and 0.5 Hz for respiration detection, and another with cut-off frequencies of 1 Hz and 3 Hz for heart rate estimation. As is well known, breathing induces small chest movements that cause variations in the radar signals reflected by the body. These variations produce harmonics that can interfere with the estimation of heart rate. Therefore, a notch filter should also be employed to remove these harmonics from the heartbeat signal. Also, range bins with an amplitude greater than thr2 are combined with those in close proximity to the target range in order to improve the accuracy of heart rate reading. In this manner, the range ambiguity is reduced as a result of the higher SNR, which smooths out the extracted phase signal and averages out any small variations in the reflected signal.

The fundamental aspect of CWT is centered around the wavelet function, a mathematical equation characterized by its localization properties in both the time and frequency domains. CWT involves systematic manipulation of the scale and translation parameters of the wavelet function across a signal. Scaling the wavelet alters its frequency, and translating it accounts for variations in time. The result of the CWT is often represented as a scalogram, which is a two-dimensional plot showing how the strength of the wavelet transform varies with time and frequency. The CWT of a signal f(t) with respect to a wavelet Ψ(t) can be expressed using the following formula:

(4) $$CWT=\;\frac{1}{\sqrt{a}}\int_{-\infty }^{+\infty }f\left(t\right)\;\Psi^{*}\left(\frac{t-b}{a}\right)\;dt$$

where Ψ is the wavelet function, and *a* and *b* are the scale and translational values, respectively. In CWT, *a* is discretized by an integer larger than one, named voice per octave. As the value of *v* increases, the discretization of the scale parameter becomes more precise. There are different types of wavelets [26] with various characteristics. In our algorithm, we use a Morlet wavelet and a generalized Morse wavelet to extract breathing and heart rate, respectively. In generalized Morse, two parameters define the characteristic of the wavelet. One is a time-bandwidth product (*P*) determining duration in time. The second is symmetry (γ) controlling the wavelet shape in time. In this algorithm, *P* and γ are 100 and 3, respectively. After applying CWT, the scale with the highest amplitude and longest duration in time is considered as the desired frequency (breathing and heart rate).

## 3. Vital Signs Algorithm Simulation

### 3.1. Equations of Transmitted and Received Signals in FMCW MIMO Radar

In an FMCW radar, a chirp starts at a certain frequency, ramps up or down at a constant rate, and then returns to its original frequency to start the next chirp. This process repeats continuously as the radar operates. The transmitted chirp has several important parameters that are carefully designed for the radar’s specific requirements. These parameters include the chirp’s bandwidth (the range of frequencies covered during each sweep), the chirp duration (the time it takes to complete one sweep), and the chirp repetition frequency (frame period).

In a MIMO radar, if the *m*th transmitter antenna location is $d_{m}=\left(m-1\right)\;d_{Tx}$ where $d_{Tx}$ is the distance between two transmitter antennas (Tx), the transmitted signal is defined as [17],

(5) $$s_{T_{m(t)}}=A_{Tm}\cos\left(2\pi f_{c}\left[t-\left(m-1\right)T_{r}\right]+\frac{\pi B}{T_{c}}{\left[t-\left(m-1\right)T_{r}\right]}^{2}+\frac{2\pi }{\lambda }d_{m}\sin\theta_{T}+\varphi_{m}\left(t\right)\right)$$

where $A_{Tm}$, $f_{c}$, $T_{c}$, and $T_{r}$ are the transmitted power, the chirp starting frequency, the chirp duration, and the switching duration between transmit antennas, respectively. *B* and λ are the chirp bandwidth and the wavelength, respectively. Also, $\theta_{T}$ is the angle of departure from *m*th transmitter antenna to target and $\varphi_{m}\left(t\right)$ is the phase noise from the *m*th transmitter. When the transmitted signal encounters a target (such as a person), part of the signal is reflected back towards the radar. This echo is received by the radar’s antenna. It is then mixed (multiplied) with the current transmitted signal, which is still being continuously transmitted by the radar. The mixing process generates a beat frequency signal as the result of the difference in frequencies between the transmitted and reflected signals. For the beat signal received at *n*th receiver antenna at location $d_{n}=\left(n-1\right)\;d_{Rx}$ where $d_{Rx}$ is the separation between two receiver antennas (Rx), the *k*th ADC sample and the *l*th chirp after I/Q sampling can be written as [17]

(6) $$\begin{array}{c} s_{B}\left(k,l\right)=A_{mn}e^{j\left\{2\pi f_{c}\left(m-1\right)T_{r}+2\pi f_{b}kT_{f}+\frac{4\pi }{\lambda }\left[R_{target}+∆R\left(lT_{s}\right)\right]\right\}}\\ \times \;e^{j\left\{\frac{2\pi }{\lambda }\left(d_{m}\sin\theta_{T}+d_{n}\sin\theta_{R}\right)\right\}} \end{array}$$

where $T_{f}$, $T_{s}$ are the fast-time and slow-time ADC sampling periods, respectively. $\theta_{R}$ represents the angle at which the target arrives at the *n*th receiver antenna. $A_{mn}$ is the received power. Also, $f_{b}$ is equal to $B\left[\left(m-1\right)T_{r}+t_{d}\right]/T_{c}$, named the beat frequency. In (6), the object range is defined by $R_{target}$ and shows the small variations due to physiological effects (∆R). By using signal processing techniques on the extracted phase of the received signal, the target’s vital signs rate can be determined.

### 3.2. Simulation

**Figure 5:**
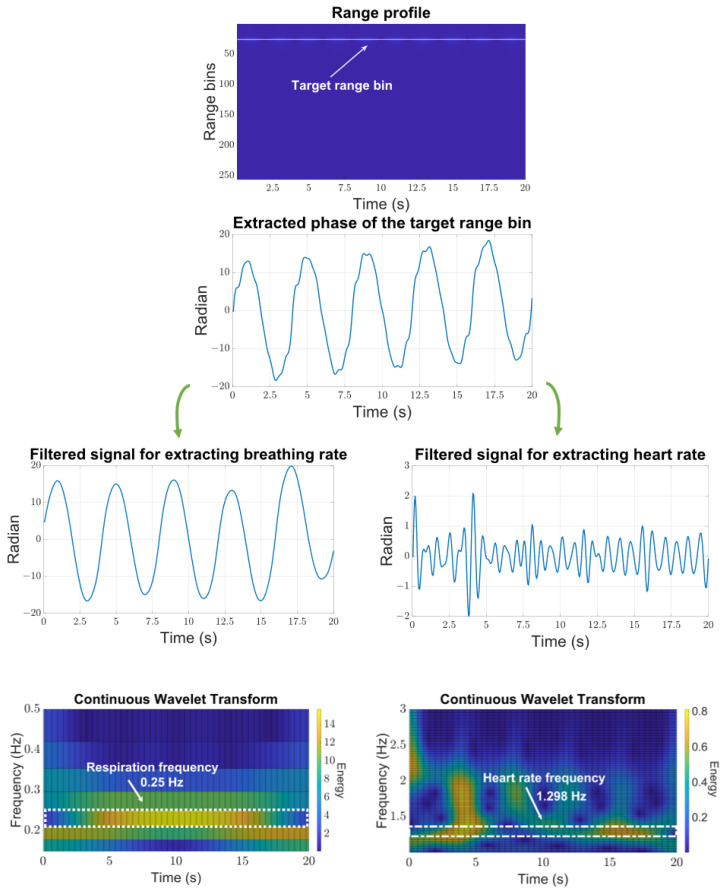
Simulation of the suggested vital sign algorithm. The bottom left and right graphs show a concentration of energy around 0.25 Hz and 1.298 Hz, which correspond to the breathing and heart rate, respectively.

To simulate the described processing pipeline, the calculation of (6) is conducted based on the parameters outlined in Table 1. Moreover, it is postulated that the individual is situated at a distance of 1.5 m. To simulate the person’s vital signs, two sine functions with amplitudes of 6 mm and 0.2 mm and frequencies of 0.25 Hz and 1.3 Hz, respectively, are taken into consideration. This choice is made due to the fact that the typical chest displacement caused by breathing and heartbeat is approximately 6 mm and 0.2 mm. To consider respiration harmonics, sine signals, characterized by frequencies extending up to eight harmonics of the breathing rate, are incorporated into the simulated vital sign signal. These sine signals have the same amplitude as the heartbeat signal. By employing bandpass filters and conducting CWT analysis, the frequencies of the vital signs can be revealed. This is illustrated in Figure 5. Within this figure, the frequency with the greatest magnitude and the longest temporal spread is deemed to represent the vital sign rate.

## 4. Preliminary Steps as Precursors to Wide Angular Vital Sign Monitoring

This section delves into the techniques of localization and beam steering, which serve as the foundation (Steps 1 and 2 in Figure 1) for the subsequent application of wide angle vital sign monitoring. While these steps are not novel in their individual application, their integration and the synergy they create are important for the robust operation of extracting vital signs across varying distances and angles.

### 4.1. Localization Technique

**Figure 6:**
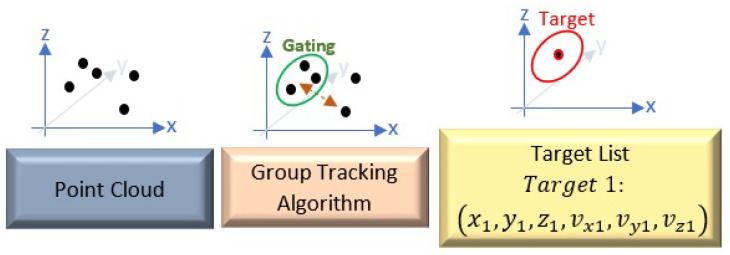
The overall localizing processing chain.

Through the analysis of the received echo signals, the radar aims to ascertain the object’s velocity, range, and direction. This is accomplished by utilizing fast Fourier transforms (FFT) on the sampled reflected signals. The range of the object is determined by performing a one-dimensional FFT on each chirp, which allows for the extraction of valuable range-related data. Additionally, the velocity of the object is estimated by applying a two-dimensional FFT on each frame, known as the 2D FFT, providing insights into the object’s speed. Furthermore, to localize a target accurately, the angle of the target should be determined, too. Since a single-input–single-output radar cannot measure this angle, several transmit and receive antennas should be used to measure the direction. Therefore, an MIMO radar is a cost-effective candidate with high anglular resolution especially at high frequencies, where antenna dimensions are compact. The direction of the object is ascertained through the application of either a three-dimensional FFT or an angle-FFT on the data collected from azimuth and elevation antennas. This step allows for the determination of the object’s orientation in three-dimensional space. The outcome of these signal processing steps is represented as a known point cloud, which offers a representation of the detected object’s characteristics. Since there are a lot of reflected echoes from different objects in the radar field of view, localizing targets is challenging. Therefore, to precisely detect the target’s points, algorithms must be implemented. The group tracking algorithm suggested by TI [25] is utilized in this paper. This algorithm involves a comprehensive search for clusters, employing an intensive examination of both Cartesian and Doppler space. Cartesian space serves as the three-dimensional domain where the point cloud data are accurately represented, providing a spatial context for the detected objects. The Doppler space relates to the frequency shift of the reflected signals, offering insights into the motion and velocity of the targets. During the cluster search stage, the algorithm focuses on identifying coherent groups of data points that exhibit close proximity in both spatial and temporal dimensions. The reasoning behind this step is to find potential clusters of signals that might correspond to objects within the radar’s surveillance area. By isolating and identifying these clusters, the radar system gains a better understanding of the environmental conditions and can initiate effective object tracking. These clusters of closely located points may indicate the presence of real-world objects, such as persons, pets, or other relevant targets. Once the clusters are successfully recognized, the next crucial step involves predicting the kinematic behavior of these clusters over time. This predictive aspect allows for the radar system to maintain a continuous and accurate track of the unique objects. The prediction is achieved by considering various factors, such as the velocity and direction of the clusters. The algorithm may also utilize any other available information about the objects being tracked, such as historical data or prior tracking results to improve the accuracy of the predictions. This sophisticated prediction mechanism enables the radar system to anticipate the future positions and trajectories of the tracked objects, thereby facilitating a proactive response to significant environmental changes. By continuously updating the tracks and adjusting the predictions based on the latest information, the radar system can provide real-time situational awareness. The processing chain for tracking and a brief explanation of some important parameters used by the algorithm are seen in Figure 6 and Table 2, respectively. Also, more details can be found in [25].

### 4.2. Mechanism of Beam Steering

MIMO configuration enhances the system’s capability to detect small-scale movements, such as chest displacement due to breathing and heartbeat. Different receiver antennas can capture the reflected signals from various spatial points, thereby increasing the overall SNR. Also, multiple transmitter antennas can be used to focus the beam, which increases the signal’s intensity on the subject, improving reflection properties. In IWR6843AOPEVM (Texas Instrument, Dallas, TX, USA), there are three Tx channels; their configuration can be found in [23]. Each Tx channel possesses a 6-bit programmable phase register with a step size of 5.625 degrees [27]. By adjusting the phase values of each Tx channel, the main beam can be steered in a specific direction. This enables the radar system to focus its energy on a particular area of interest, increasing the accuracy and effectiveness of the transmitted signals. In detecting vital signs, focusing the beam is also effective in distinguishing associated range bins (the target’s range bins) from other nearby clutter based on its energy, and it allows for us to monitor physiological parameters in any direction relative to the radar [21]. The array factor and target angle are used to calculate the amount of phase value to be programmed to each Tx channel. In the case of *N* Tx channels, with Tx1 serving as a reference, the distance between each antenna in the array and Tx1 is known during manufacturing ($d_{N}$). The phase signal for each transmitter channel (TxN) is calculated as

(7) $$\begin{array}{c} \left[\begin{array}{cccc} \varphi_{1} & \varphi_{2} & \cdots & \varphi_{N} \end{array}\right]=\left[\begin{array}{cccc} 0 & 2\pi \frac{d_{2}}{\lambda }\sin\theta & \cdots & 2\pi \frac{d_{N}}{\lambda }\sin\theta \end{array}\right] \end{array}$$

where λ is the wavelength and θ is the direction in which the beam should be pointed. Since the step phase size is 5.625 degrees, the final phase value entered into the register is

(8) $$\bar{\varphi }=\left\lfloor \frac{\left[\begin{array}{ccc} \phi_{1} & \cdots & \phi_{N} \end{array}\right]}{5.625}\right\rfloor$$

## 5. Measurement Results and Discussion

The proposed approach was evaluated using a mm-wave radar (IWR6843AOPEVM, Texas Instrument, Dallas, TX, USA), operating between 60 GHz and 64 GHz. According to [23], for safety reasons, it is recommended to keep a minimum distance of 20 cm between users and the radar while it is in operation. The radar was connected to a laptop using UART for data communication. To measure the power consumption of the radar, we used a USB volt and ampere meter, specifically JT-UM25C, DollaTek, 250 King’s Road, Hongkong. The maximum power consumption was 2.2 Watt while the radar was operating. Since the group tracking algorithm is handled by the radar’s internal processor, all data, sent out on UART, are the person’s location and velocity and the range bins. As seen in Figure 2, the range bins associated with receiver Channels 1 and 3 are mainly sent due to the constraint of the UART data rate. It should be noted all transmitter antennas send chirps simultaneously to realize a beam focused on the person’s location. Therefore, it is not necessary to use all range bins corresponding to all transmitters. The algorithm for calculating vital signs was executed on a laptop using the MATLAB (R2021b) platform. There are two configurations for the radar to detect the person’s coordinates and vital signs. Table 3 shows the configurations. After localizing, when the person is static (according to their velocity), the radar is reconfigured automatically and focuses its beam to start monitoring physiological parameters.

**Figure 7:**
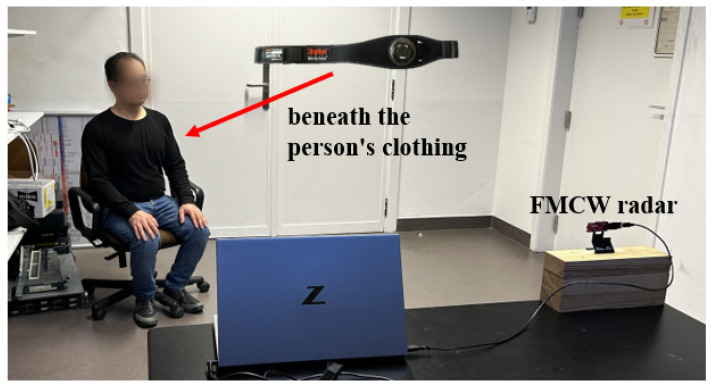
The experimental setup. A participant seated in a chair positioned within a designated area after entering the room and walking.

In our experiment scenario, a person walked through a room with a dimension of 3 × 3 m^2^ and was asked to sit at a desired place with an angle between −45 and +45 degrees and a distance of up to 2.3 m to the radar. The experiment was conducted under a controlled scenario (the person remained stationary, and their chest was toward the radar). The range bins were collected for a 20 s sliding window with a 15 s overlap. The values of thr1 and thr2 in Figure 3 are 700. We employed a chest-worn sensor, namely the Zephyr Bioharness 3 (Zephyr Technology, Boulder, CO, USA), as the ground truth. The reference sensor’s output was transmitted to a laptop via Bluetooth, enabling us to monitor both the extracted vital signs and reference values simultaneously in an online mode. The measurement was repeated for six participants at different angles and distances. The minimum time of each measurement was about 100 s. Also, the most recent four outcomes were combined to achieve a more uniform graphical representation. Figure 7 and Table 4 illustrate the testbed and measurement results, respectively. In Table 4, root mean square error (RMSE) and mean absolute error (MAE) quantify the mean errors between the estimated results of the radar and the results obtained from the contact sensor for different positions of participants. It is worth mentioning that the distance and angle were determined based on the group tracking algorithm results. Another aspect that should be considered is SNR. Our observations indicate that SNR for each point within the point cloud is approximately 18 dB. Furthermore, when focusing on vital sign detection, the SNR values typically range from 12 dB to 15.3 dB. In the evaluation of the measured respiration rate across different positions, it was observed that the lowest RMSE and MAE were recorded as 0.39 and 0.35, respectively. Conversely, the highest values for RMSE and MAE were found to be 1.09 and 0.83, respectively. Shifting our focus to the measured heart rate, it was noted that the maximum RMSE and MAE values were 9.49 and 9, respectively, whereas the corresponding minimum values were 3.24 and 2.66 for RMSE and MAE, respectively. The measured results highlight the efficacy of the proposed algorithm. Notably, there was an observed enhancement in both breathing and heart rate metrics when compared to [21]. Specifically, the improvement in breathing rate was as high as 92%, while heart rate metrics showed a notable increase of 58%. These results validate the effectiveness of the proposed algorithm.

**Figure 8:**
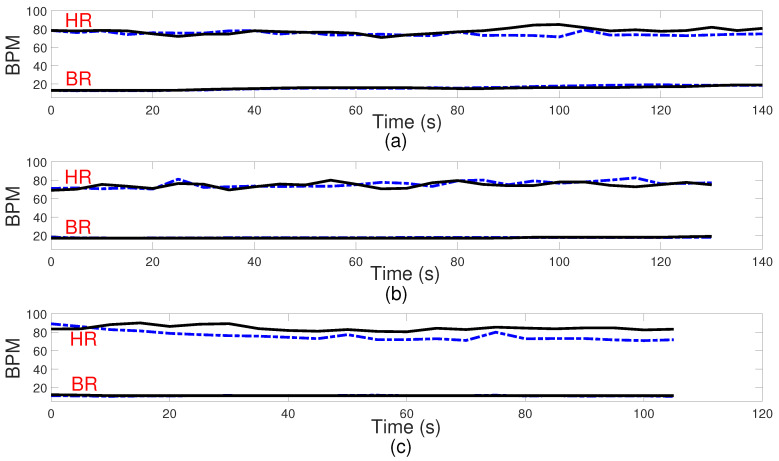
Respiration (BR) and heart rate (HR) in beats per minute (BPM). Comparison between the algorithm results and ground truth sensor for some participants. Subject 1 at the distance of 1.84 m and the angle of −26.8 degrees (**a**), Subject 2 at the distance of 1.28 m and the angle of 19.2 degrees (**b**), Subject 3 at the distance of 1.93 m and the angle of −44.8 degrees (**c**). Dashed line in blue: measured results; solid line in black: ground truth.

**Figure 9:**
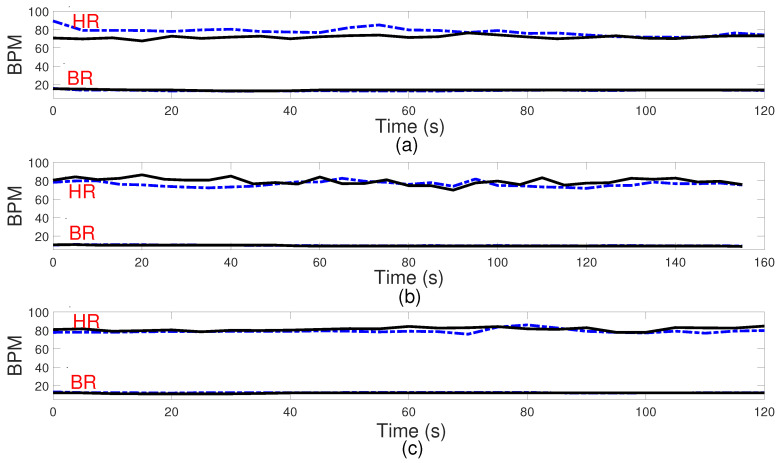
Respiration (BR) and heart rate (HR) in beats per minute (BPM). Comparison between the algorithm results and ground truth sensor for some participants. Subject 4 at the distance of 2.25 m and the angle of 19.93 degrees (**a**), Subject 5 at the distance of 1.87 m and the angle of −34.16 degrees (**b**), Subject 6 at the distance of 1.15 m and the angle of 37.78 degrees (**c**). Dashed line in blue: measured results; solid line in black: ground truth.

**Figure 10:**
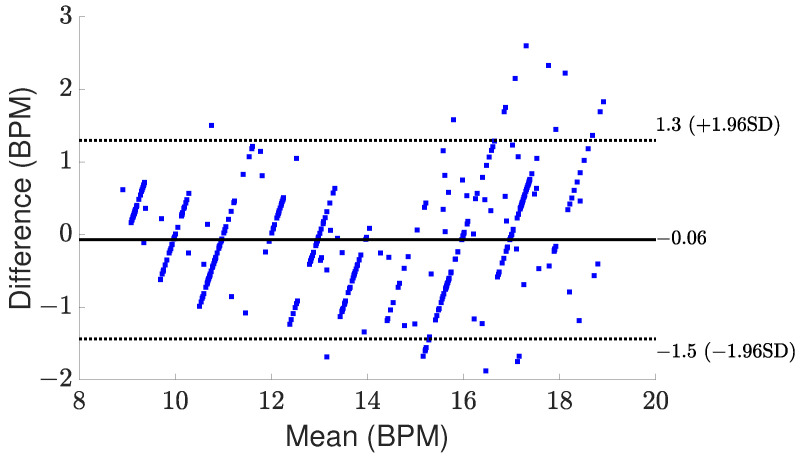
Bland–Altman plot for the measured respiration rate.

**Figure 11:**
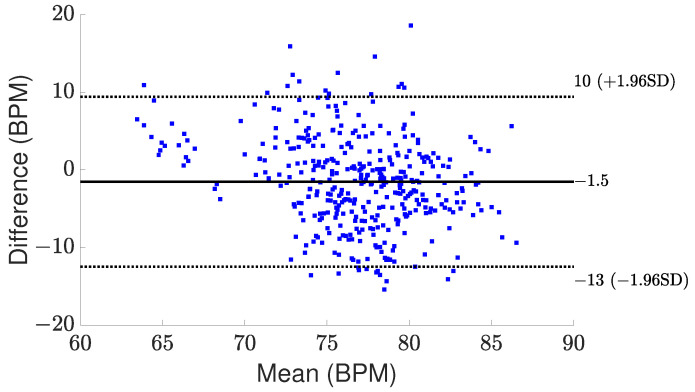
Bland–Altman plot for the measured heart rate.

Figure 8 and Figure 9 present comparisons between the estimated breathing and heart rates and the corresponding values acquired from the ground truth sensor for subjects in different positions. Although a significant association is seen between the actual values and the recorded outcomes for respiratory rate, there exists a greater mismatch between the derived heart rate and the benchmark sensor. The observed difference can be attributed to the reflected signal originating from the internal organ (heart), which demonstrates reduced amplitude and experiences greater attenuation throughout propagation. One additional element that potentially complicates the detection of heart rate is the introduction of phase noise in the RF front end of the radar. This phase noise in the RF front end of the radar can lead to errors in the measurement of heart rate, as it adds random phase variations to the received signals. These fluctuations can cause signal distortion, making it challenging to calculate a more accurate heart rate. However, amplifying the signal power, employing additional transmitting antennas to concentrate the signal more precisely on the subject, and utilizing more receiver antennas to capture a broader range of signals can be proposed to enhance the detection of these subtler heart-induced displacements. To assess agreement between extracted results and reference values graphically, Bland–Altman plots of breathing and heart rates are illustrated in Figure 10 and Figure 11, respectively. The horizontal and vertical axes show the mean and difference values for each pair of measurements. As seen, the average difference is −0.06 BPM with the limits between −1.5 BPM and 1.3 BPM for breathing rate. Regarding heart rate, the average error is −1.5 BPM with a range from −13 BPM to +10 BPM.

Table 5 presents a comparative analysis between this work and other published studies. Our research distinguishes itself by emphasizing the tracking of vital signs from a wide range of angles. This is achieved by the utilization of a commercially available FMCW radar, while simultaneously addressing the aspects of real-time monitoring and two-dimensional localization. Nonetheless, monitoring vital signs in a wider angular range is limited to the number of transmitter antennas available for beam steering, their spatial configuration, and the degradation of signal quality at wider angles, which also depends on the receiver antenna configuration. Despite these limitations, it is important to acknowledge that, although the study may not achieve the same level of accuracy as some existing works in the field, our approach brings valuable contributions to the domain of vital sign monitoring. Our methodology prioritizes the practical aspects and real-world scenarios, which play an important role in realizing healthcare in smart homes. By adopting this approach, we strike a balance between accuracy and wide angular monitoring and practical utilities, offering a novel perspective.

## 6. Conclusions

This work introduces a method for monitoring vital signs in a flexible angular position. The approach combines a localization algorithm, a beamforming technique, and the CWT method to efficiently monitor a person’s vital signs. The utilization of a group tracking algorithm enables the detection of a static person’s location, providing both position and velocity data. When the person is stationary, the radar automatically reconfigures, focusing its beam to extract respiration and heart rate. Notably, the group tracking algorithm and raw signal processing are efficiently managed by the radar’s internal processor, with data transmission to a laptop via UART for further vital sign extraction processing in a real-time manner. This capability is pivotal for realizing healthcare applications in smart homes. Furthermore, our approach introduces a multifunctional system, which can leverage the detected person’s location for automated control systems like lighting and temperature in smart homes. In the experiments, a compact and low-power FMCW radar, IWR6843AOPEVM (Texas Instrument, Dallas, TX, USA), is used to validate the suggested idea. Measured results within a distance range of 1.15 to 2.3 m and an angle between −44.8 and 44.8 degrees indicate effective performance, with a maximum and minimum RMSE of 9.49 BPM and 3.24 BPM for heart rate, and 1.09 BPM and 0.39 BPM for breathing rate, respectively. Bland–Altman plots show average errors of −0.06 BPM for breathing rate and −1.5 BPM for heart rate. While the experiments demonstrate the viability of the proposed approach, we acknowledge limitations. Future work will address challenges such as monitoring in noisy environments (e.g., body movement, talking, walking) and considering multiple people. Additionally, we will explore the monitoring of vital signs in different orientations, not limited to various angles but also including front/back positions with respect to the radar, enhancing the robustness and applicability of our system.

## Figures and Tables

**Table 1:** Parameters used for simulation.

Parameters	Value
$f_{c}$	60 GHz
ADC sampling frequency	$5\times 10^{6}$ per second
Slow-time sampling frequency (frame period)	20 Hz (50 ms)
Number of transmitter antennas	1
Number of receiver antennas	2
The chirp bandwidth	2.5 GHz
Number of chirps	12
Separation between receiver antennas	λ
Number of ADC samples	256

**Table 2:** Group tracking parameters.

Parameters	Description
Environmental Factors (Xmin, Xmax, Ymin, Ymax, Zmin, Zmax)	Determining the physical dimensions within which the tracker functions
Gating (width, depth, height, velocity)	Defining the maximal volume and velocity of an object being tracked and is used to associate the points with existing tracks
Allocation (SNR thr, velocity thr, points thr, distance thr, diffVelocity thr)	To detect a new target in the scene when the points do not correspond to existing track by defining thresholds (thr) for SNR, velocity, number of points, the distance between points, velocity difference between points

**Table 3:** Group tracking parameters, and FMCW radar configuration for localization and vital sign detection.

Radar Configuration						
	**Localization**			**Vital Sign**		
No. of Transmitter Antennas	3					
No. of Receiver Antennas	4					
Frequency Sweep Rate	50 MHz/μs					
Frame Period	100 ms			50 ms		
No. Chirps	64			12		
Range Resolution	0.056 m			0.046 m		
Velocity Resolution	0.1528 m/s			3.24 m/s		
**Group Tracking Parameters**						
Gating Parameters	Gain	Width	Depth	Height	Velocity	
	4	2 m	2 m	2 m	10 m/s	
Allocation Parameters	SNR thr	Obscured SNR thr	Velocity thr	Points thr	max Squared Distance thr	maxVelocity thr
	200	155	0.5 m/s	10	2 m^2^	2 m/s

**Table 4:** Measurement accuracy of respiration and heart rate estimation at various distances and angles.

Subjects	Distance (m)	Angle (Degree)	Respiration Rate		Heart Rate	
			MAE	RMSE	MAE	RMSE
1	1.58	25.7	0.76	0.93	5.52	6.78
	1.84	−26.8	0.83	1.09	3.74	4.96
2	1.4	−24.6	0.72	0.85	4.52	6.19
	1.28	19.2	0.5	0.57	3.05	3.89
3	2.3	40	0.49	0.57	7.29	8.14
	1.93	−44.8	0.35	0.45	9	9.49
4	1.64	19.75	0.4	0.48	3.95	4.69
	1.91	−7.97	0.63	0.83	6.13	7.15
	2.25	19.93	0.48	0.61	5.95	7.29
5	1.87	−34.16	0.35	0.39	4.54	5.41
	1.69	35.2	0.39	0.43	4.24	5.3
6	1.75	−24.09	0.7	0.78	3.15	3.5
	1.15	37.78	0.5	0.62	2.66	3.24
	1.75	−34	0.67	0.82	2.66	3.98

**Table 5:** Comparison table.

	Distance (m)	Angle (degree)	ERROR			Radar	Online Monitoring	2D Localization
			BR	HR				
			MAE	RMSE	MAE			
[12]	1–2.5	0	-	<6	-	IWR1642	Potential	No
[10]	0.5–2.5	0	-	<2.3	-	IWR1843	Potential	No
[9]	1	0	-	-	-	Custom-designed	Yes	No
[16]	1–1.2	−30–+30	<≈0.6	-	≈6	Custom-designed	Yes	No
[13]	2	0	-	<3.33	-	Custom-designed	-	No
This work	1.15–2.3	−44.8–+44.8	<0.83	<9.49	<9	IWR6843AOPEVM	Yes	Yes

## Data Availability

The original contributions presented in the study are included in the article, further inquiries can be directed to the corresponding author.
